# Understanding the Influence of Rock Content on Streaming Potential Phenomenon of Soil–Rock Mixture: An Experimental Study

**DOI:** 10.3390/s22020585

**Published:** 2022-01-13

**Authors:** Xin Zhang, Mingjie Zhao, Kui Wang

**Affiliations:** 1 Key Laboratory of Hydraulic and Waterway Engineering of the Ministry of Education, Chongqing Jiaotong University, Chongqing 400074, China; zx@mails.cqjtu.edu.cn (X.Z.); 990000980717@cqjtu.edu.cn (M.Z.); 2 Engineering Research Center of Diagnosis Technology and Instruments of Hydro-Construction, Chongqing Jiaotong University, Chongqing 400074, China

**Keywords:** soil–rock mixture, streaming potential coupling coefficient, streaming potential, rock content

## Abstract

To applicate streaming potential phenomenon to study the seepage feature in the soil–rock mixture (SRM), research on the variation in the streaming potential phenomenon of SRM is the precondition. This paper deals, in assistance with the streaming potential test apparatus, with the streaming potential effect response of SRM subjected to different rock contents. The test results show that when the rock content increases from 10% to 30%, the streaming potential coupling coefficient increases with the increases in rock content at 85% compactness and 0.01 mol L^−1^ salinity. When the rock content is more than 30%, the streaming potential coupling coefficient decreases with the increases in rock content. As the rock content increases, the permeability coefficient has a negative correlation with the streaming potential coupling coefficient. The streaming potential increases first and then goes down with the increases in rock content, and the streaming potential decreases significantly when the rock content exceeds 50%. The findings indicate that the rock content is the key structural factor that restricts the streaming potential phenomenon of the SRM.

## 1. Introduction

Excavation and blasting are common during the construction of water conservancy facilities. The resulting SRM materials are used in large quantities, which are normally used as filling materials in dam projects. The SRM is a porous medium composed of rock with great strength and size, fine-grained soil, and pores [1]. It is essential to understand the seepage behavior of SRM for the sake of dam safety evaluation and disaster warning, and ensuring the long-term normal operation of the dam. The streaming potential phenomenon shows a good performance in tracking water flow of porous media [2,3,4,5,6], and the study of streaming potential phenomenon in SRM is helpful to further capture the seepage characteristics of SRM. However, the underlying physics of the streaming potential effect of SRM have not been well recognized.

A large number of authors have studied the influence of rock content on the permeability coefficient of SRM by different methods. SRMs consisting of a mixture of soil and rock of different materials were tested in the laboratory, which proved that the rock content had a significant effect on the permeability coefficient of the SRM [7,8,9,10]. The numerical methods successfully reproduced the influence of rock content on the seepage characteristics of SRM [11,12]. The development of the model for the permeability coefficient of the SRM further revealed the quantitative relationship between the rock content and the seepage behavior [13].

A considerable amount of literature has been published on the streaming potential phenomenon. The generation of streaming potential is related to the distribution and movement of excess counterions in the diffusion layer. The other counterions in the stern layer are confined to the surface of the charged porous medium. The diffusion layer and the stern layer form an electric double layer. The flow water carries the excess counterions in the diffusion layer to form a streaming current and an electrical potential. This electrical potential drives the movement of ions in the solution to generate a conduction current. When the streaming current is balanced with the conduction current (*j* = 0), the obtained potential is the streaming potential [14,15,16,17]. The streaming potential coupling coefficient is determined by the relationship between streaming potential gradient ∇*U* (V) and pressure gradient ∇*P* (Pa):

(1) $$C={\frac{\nabla U}{\nabla P}|}_{j=0}$$

The Helmholtz–Smoluchowski (HS) equation reveals the mechanism of streaming potential phenomenon in porous media [18,19]:

(2) $$C=\frac{\varepsilon \zeta }{\mu \sigma_{f}}$$

where *C* (V Pa^−1^) is the streaming potential coupling coefficient, *ɛ* (F m^−1^) is the dielectric permittivity, *μ* (Pa s) is the dynamic viscosity, *σ_f_* (S m^−1^) is the fluid conductivity, and *ζ* (V) is the zeta potential. The HS equation does not consider the medium structure, so it is widely used in the study of streaming potential phenomena of different medium materials (when surface conductivity is not important compared to solution conductivity). Salinity has also become the main influencing factor of HS equation [20,21,22,23]. Many authors have developed the HS equation by considering the surface conductivity, and they found that at low concentrations and low permeability, the surface conductivity dominates the electrical conduction, and the streaming potential coupling coefficient is related to the structure of the medium [24,25,26,27]. The modified HS equation is given:

(3) $$C=\frac{\varepsilon \zeta }{\mu \sigma_{rw}F}=\frac{\varepsilon \zeta }{\mu (\sigma_{f}+\sigma_{s})}$$

where *σ_S_* (S m^−1^) is the surface conductivity, *σ_rw_* (S m^−1^) is the electrical conductivity of the saturated sample, and *F* is the intrinsic formation factor of the sample. As noted above, when using HS equation to study the streaming potential effect of medium, it is necessary to consider the surface conductivity. It is convenient to study the streaming potential effects of the medium once the surface conductivity is neglected. If the surface conductivity is the main contribution to the conductivity of the medium, the intrinsic formation factor of the medium at high salinity needs to be measured, and the research process will become complicated.

The effective excess charge density approach is an alternative method to express the streaming potential phenomenon in porous media. The streaming potential effect is attributed to the transport of effective excess charge in the diffusion layer [28,29]. The effective excess charge density is used to calculate the streaming potential coupling coefficient [30].

(4) $$C=\frac{Q_{v}k}{\mu \sigma }$$

where $Q_{v}$ (C m^−3^) is the effective excess charge density, *k* is the permeability (m^2^), *μ* (Pa s) is the dynamic viscosity, and *σ* (S m^−1^) is the electrical conductivity of porous media. In order to predict the streaming potential coupling coefficient, it is essential to obtain the effective excess charge density. Previous studies have shown an empirical relationship between effective excess charge density and permeability [3,4]. Recently, the analytical model from Guarracino and Jougnot [31] further determines the quantitative relationship between effective excess charge density and permeability. This study further lays a theoretical foundation for the application of effective excess charge density approach. When the effective excess charge density is used to study the streaming potential effect, the surface conductivity does not need to be considered. At the same time, it is directly related to the medium structure parameters, and the application range becomes wider. However, when the solution concentration changes significantly, the effective excess charge density cannot be accurately obtained through the empirical relationship with permeability. At this time, Guarracino and Jougnot’s [31] analytical model needs to be used, which means that more parameters need to be obtained, which makes the research inconvenient.

The proposed effective excess density charge approach promotes the use of the self-potential method. The self-potential method is used to record the electric field produced by streaming potential in real time. This method is cost-effective and has a wide measuring range, quick response, and high sensitivity. Much of the available literature on applying the self-potential method deals with the question of detecting the leakage of embankment dams. Spatial distribution of obtained self-potential signal was used to delineate leakage in an earth rock dam in the early application [32]. With the development of effective excess charge density approach, the result of forward calculation was more intuitive and easier to interpret [33,34,35]. The further development of the inversion algorithm, namely the self-potential method, has realized the quantitative analysis of the leakage path of the embankment dam, which is of great significance for us to assess the safety of the dam [36,37,38]. In the future, the use of statistical principles and considering the heterogeneity of embankment dam materials will further improve the ability of the self-potential method to quantitatively analyze the physical parameters of the leakage path [39,40,41].

The aforementioned studies show that the critical role played by the rock content on the seepage features of SRM. Using streaming potential phenomenon to study the seepage characteristics of SRM can broaden the understanding of the problem. Although the self-potential method has been widely used in detecting the leakage in embankment dams, the mechanism of streaming potential phenomenon of SRM is rarely studied. The research on the influence of rock content on the streaming potential effect of SRM will promote the further application of the self-potential method in the embankment dam.

In this study, our goal is to reveal the effect of rock content on the streaming potential phenomenon in SRM. We designed five SRMs with different rock contents and measured the potential of the SRMs at different water levels. The correlation between the streaming potential coupling coefficient and the permeability coefficient are analyzed. The results lay a foundation for monitoring the internal erosion of embankment dams by self-potential method.

## 2. Experimental Methodology

**Figure 1:**
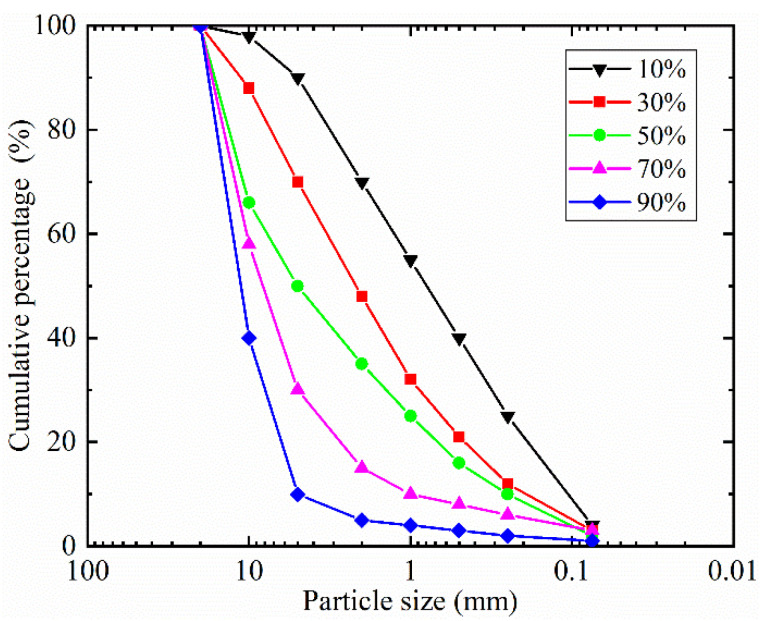
Cumulative gradation curve of different rock contents.

The material of the SRM is the typical weathered broken argillaceous rock and comes from the subgrade of sections K1 + 290~K1 + 350 and K0 + 720~K0 + 800 of Xuetang Road, Chongqing, China. In this paper, the threshold value of soil and rock is taken as the 5 mm adopted by Zhou et al. [13], such that particles greater than 5 mm are rock and those less than 5 mm are soil. The maximum particle size was less than 20 mm. SRMs with rock content of 10%, 30%, 50%, 70%, and 90% were designed (the ratio of the mass of the rock to the mass of the SRM was defined as the rock content), and the grading distribution curves are shown in Figure 1. We can judge the grading characteristics by using the nonuniform coefficient *C_u_* = *d*_60_/*d*_10_ and the curvature coefficient *C_c_* = *d*_30_ · *d*_30_/(*d*_10_ · *d*_60_). (*d*_10_, *d*_30_ and *d*_60_ denote the particle sizes of 10%, 30% and 60% of the total mass on the cumulative gradation curve, respectively [42].) *C_u_* ≥ 5 and *C_c_* = 1–3 are thought to be well-graded soils; if these two conditions cannot be met at the same time, the soil is considered poorly graded. The gradation with 10% and 90% rock content are poorly graded, and the gradations with 30%, 50%, and 70% rock content are well graded (see Table 4 in [43]). The X-ray diffraction tests indicated that the weathered broken argillaceous rock consist of quartz (48.8%), illite (22%), albite (17.9%), kaolinite (2.7%), chlorite (5.5%), calcite (1.8%), and hematite (1.2%).

**Figure 2:**
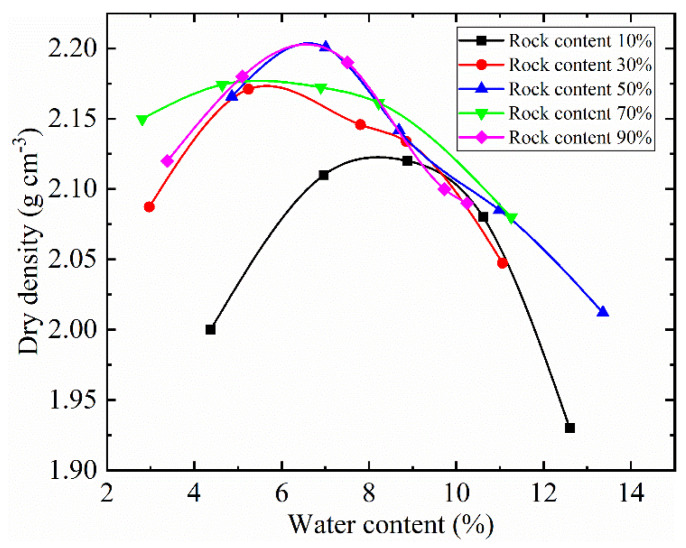
Dry density and water content of SRM with different rock content.

To ensure the same degree of compaction (85%) of the SRM with different rock content in the preparation, we adopted the following protocol. The maximum dry density and optimal water content were determined by the standard compaction test method (see Figure 2). The dry density was calculated by the following equation [44]:

(5) $$D=\frac{\rho_{d}}{\rho_{md}}$$

where *D* is the degree of compaction, *ρ_d_* (kg m^−3^) is the dry density, and *ρ_md_* (kg m^−3^) is the maximum dry density. Next, we used dry density and optimum moisture content to obtain wet density:

(6) $$\rho_{d}=\frac{\rho_{w}}{\left(1+w\right)}$$

where *ρ_w_* (kg m^−3^) is the wet density and *w* is the optimum moisture content. Finally, the mass of SRM with compactness of 85% was obtained by the following equation:

(7) $$M_{\mathrm{SRM}}=\rho_{w}\times V_{f}$$

where *M*_SRM_ (kg) is the mass of SRM and *V_f_* (m^3^) is the volume of the filling area.

At the beginning of the experiment, the SRM was saturated with 0.01 mol L^−1^ NaCl solution. We used the water content sensors to monitor the saturation of SRM. The SRM remained in the solution until the salinity on both sides was balanced. The pH of the solution was recorded by AZ-86031 multifunctional water quality detector and varied between 6 and 8 pH units.

**Figure 3:**
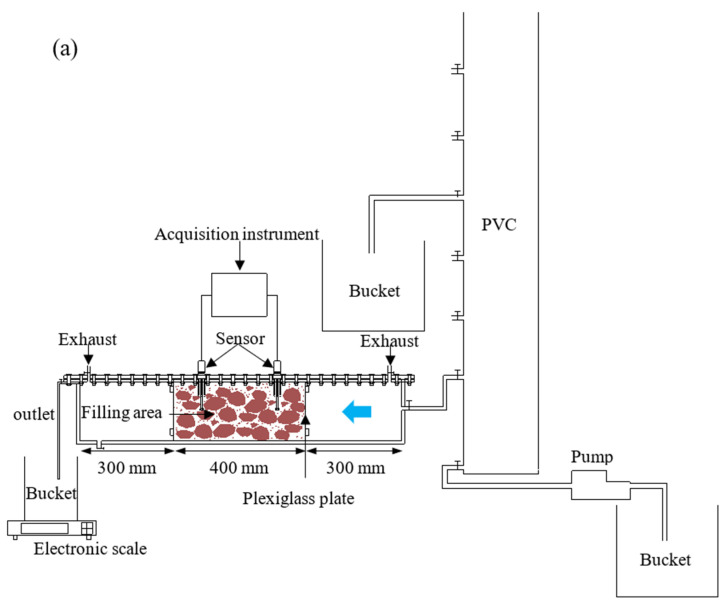

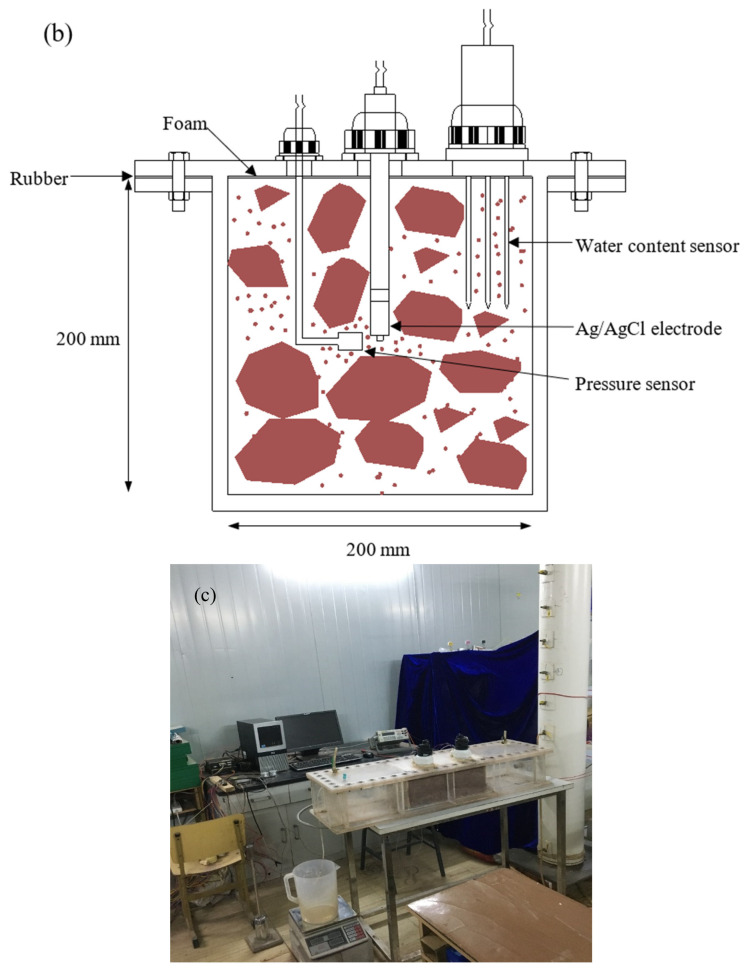
Sketch of the test apparatus. (**a**) The apparatus consists of tank (filling area, plexiglass plate, exhaust, outlet), PVC pipe (pump, bucket), and data acquisition system (sensor and acquisition instrument), the blue arrow represents the direction of the water flow. (**b**) Cross section through the sensor (water content sensor, Ag/AgCl non-polarizable electrode, pressure sensor, rubber, and foam). (**c**) The photo of testing apparatus.

Figure 3 shows the schematic diagram of the testing apparatus. The water tank was composed of plexiglass plates with holes, exhaust, and outlet. We compacted the SRM between two plexiglass plates (400 mm × 200 mm × 200 mm). We used the pressure generated by the fixed upper cover to squeeze the foam board on the SRM to prevent water flow. The CYG1145 pressure sensors, Ag/AgCl non-polarizable electrodes, and water content sensors were arranged on two sections, respectively. We used a computer to control DH3821 to measure pressure. The potential was measured by a DM3058 multi-function digital multimeter. The PVC pipe provided water pressure for the experiment. The water pressure was adjusted at 20 cm intervals within the range from 20 to 100 cm. The volume of water flowed through the SRM was obtained by the electronic scale.

CYG1145 pressure sensor has a measurement range of −50 to 50 kPa, a measurement accuracy of 0.5, a compensation temperature range of −10 to 60 °C, an operating temperature range of −40 to 80 °C, and an international standard signal 0–5 V or 4–20 mA output. In this experiment, a five-point calibration is used for the pressure sensor to ensure a good linear relationship between the parameters. The Ag/AgCl non-polarizable electrode is immersed in saturated potassium chloride solution, and the bottom is in contact with the medium through porous ceramics, which can reduce electrolyte loss. Before each use, the electrodes should be soaked in saturated potassium chloride solution for 2 h to ensure the same concentration of potassium chloride solution in the two electrodes and reduce the potential difference between the two electrodes. The measurement range of the water content sensor is 0 to 100%, the measurement accuracy is ±2%, the probe length is 78 mm, the diameter is 4 mm, and the measurement area is a cylinder with a diameter of 7 cm and a height of 7 cm centered on the central probe.

The test procedure was as follows: the initial voltage *U*_1_ (mV) and the hydraulic head difference *H*_1_ (cm) of the SRM were recorded, and then a sudden hydraulic head was applied in the upstream tank; we obtained the potential *U*_2_ (mV) and the hydraulic head difference *H*_2_ (cm) until a steady state was reached. The streaming potential was calculated by ∆*U* = *U*_2_ − *U*_1_ (mV), and the hydraulic head difference was obtained by ∆*H* = *H*_2_ − *H*_1_ (cm). In turn, the streaming potential and the hydraulic head difference of five different hydraulic head were measured. The streaming potential coupling coefficient was equal to the slope of the fitting line between the streaming potential and the hydraulic head difference.

The permeability coefficient was obtained by recording the volume of the solution during a specific period of time. Permeability can be calculated by permeability coefficient:

(8) $$K=\frac{\rho_{w}gk}{\mu }$$

where *K* (m s^−1^) is the permeability coefficient, *ρ_w_* (kg m^−3^) is the density of water, *g* (N kg^−1^) is the acceleration, and *k* (m^2^) is the permeability. We arranged stainless steel electrode net on both sides of the plexiglass plate and obtained the conductivity of the SRM in 0.01 mol L^−1^ and 1 mol L^−1^ solutions with a two-electrode device, respectively. The material properties of SRM were shown in Table 1.

## 3. Results

### 3.1. Streaming Potential Phenomenon

**Figure 4:**
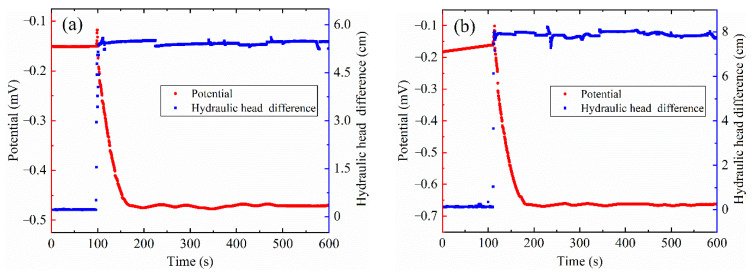
The change of potential and hydraulic head difference under different hydraulic head at 85% compaction and 10% rock content; (**a**) 20 cm hydraulic head; (**b**) 40 cm hydraulic head. The red circle symbols represent the potential, and the blue square symbols represent the hydraulic head difference.

Figure 4 shows typical variation of the potential and the hydraulic head difference for the SRM with the 85% compaction and 10% rock content. The potential and hydraulic head difference of the SRM approach a constant value before water is injected. A sudden hydraulic head is applied in the upstream tank, the brine flows through the sample. There is a hydraulic head difference fluctuations associated with the injection of the water, and then the hydraulic head difference maintains a constant value. The potential changes with hydraulic head difference. A large potential moves up with the hydraulic head difference fluctuations, and then the potential changes in the opposite direction until stable potential is observed. As the hydraulic head rises, the magnitude of the hydraulic head difference and potential increase at the same time.

### 3.2. Streaming Potential Coupling Coefficient with Different Rock Content

**Figure 5:**
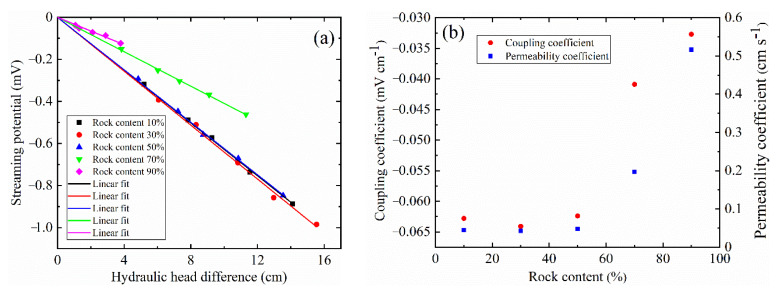
The results of streaming potential coupling coefficient and permeability coefficient with different rock contents. (**a**) Streaming potential and pressure difference. The streaming potential coupling coefficient is determined by the slope of the regression line between streaming potential and pressure difference. (**b**) The variation of streaming potential coupling coefficient and permeability coefficient.

Figure 5a shows the variation of streaming potential with hydraulic head difference. At rock contents between 10% and 50%, the change in the streaming potential is small. The value of streaming potential decreased rapidly, particularly when the rock content exceeds 50%. Figure 5b shows that the streaming potential coupling coefficient is determined by the slope of the linear regression of streaming potential against hydraulic head difference. The correlation coefficients of fitting lines with rock content of 10%, 30%, 50%, 70% and 90% are 0.999, 0.994, 0.998, 0.998 and 0.960, respectively. The magnitude of the streaming potential coupling coefficient increase first and then decrease with the increase in the rock content. At the rock content of 30%, a larger value is obtained. The rock content has a significant impact on the streaming potential coupling coefficient when the rock content is increased from 50% to 90%. The permeability coefficient varies inversely with the streaming potential coupling coefficient (see Figure 5b).

### 3.3. Effective Excess Charge Density and Rock Content

**Figure 6:**
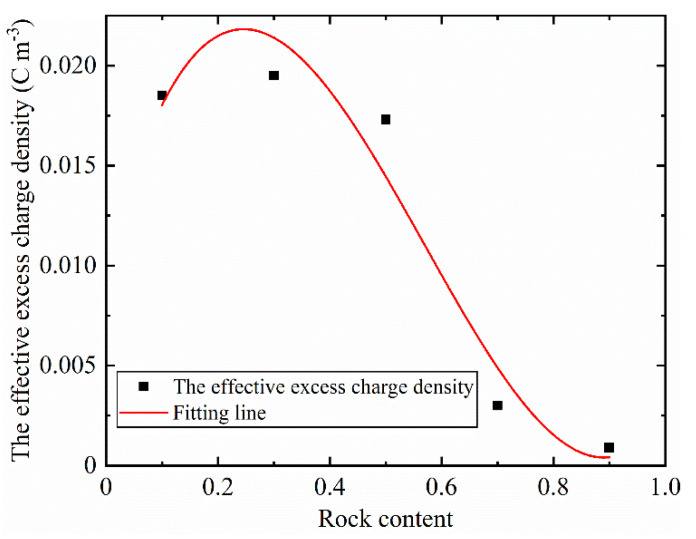
The empirical relationship between effective excess charge density and rock content.

Figure 6 shows the empirical relationship between rock content and effective excess charge density,

(9) $${\hat{Q}}_{v}=0.01007+0.10532C_{R}-0.2733C_{R}^{2}+0.16042C_{R}^{3}$$

where *C_R_* is the rock content. The correlation coefficient between polynomial and data is 0.952. The effective excess charge density first increases and then decreases with the rock content. This phenomenon can be explained by the fact that the effective excess charge density is inversely proportional to the permeability [29]. Permeability decreases first and then increases with rock content. Therefore, the effective excess charge density shows an opposite trend.

**Figure 7:**
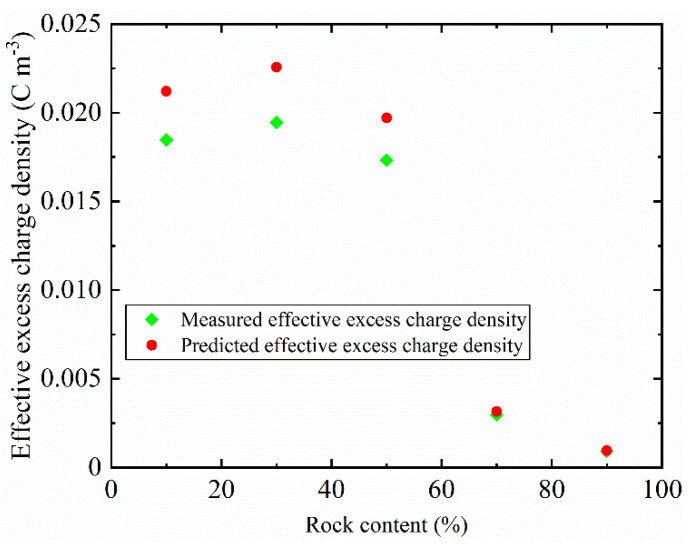
Analytical solution of effective excess charge density compared with the experimental measurements value.

We compared the analytical solution of the effective excess charge density with the obtained value of the experiment (see Figure 7). The analytical model proposed by Guarracino and Jougnot [31] reproduces the experimental data very well, which verifies the reliability of our experiment. This model shows better prediction accuracy in high rock content. This may be related to the applicable conditions of the thin electric double layer of the model. The pore radius is larger than the thickness of electric double layer in the SRM with high rock content. The fractal characteristics of porosity are considered in the analytical model, but there is no distinction between porosity and effective porosity. Because there is no water flow in the closed pore, it does not contribute to the effective excess charge density, which may lead to the measured effective excess charge density are smaller than the predicted effective excess charge density.

## 4. Discussion

### 4.1. Pressure Front and Potential

Here, the potential fluctuates upward and then drops down with the change of hydraulic head. This phenomenon is different from what was previously reported, namely that the potential changes in one direction [16,21,45] because our electrodes are placed in the middle of the sample instead of both ends. There is a corresponding relationship between the pressure front and the change in potential. The reference electrode is connected to the negative terminal of the multimeter. Under the action of water pressure, the excess cations within the diffuse layers migrate to the electrode on the upstream side. The potential’s moving up arises from the accumulation of excess positive charge around this electrode. The more excess cation along the sample move to the reference electrode with the brine flow to the downstream, the more excess positive charge gather around the reference electrode, at which time the potential goes down. According to the corresponding relationship between the pressure front and the change of potential, we can grasp the seepage pressure distribution inside the dam when the water level rises rapidly, which is helpful in order to realize the accurate description of the seepage field distribution characteristics and improve the early warning ability of emergency.

### 4.2. Influence of Rock Content on Permeability Coefficient

In this paper, we show that the permeability coefficient of the SRM is slightly smaller at the rock content 30% and increases rapidly when the rock content exceeds 50% (see Figure 5b). We compare previous studies against the results obtained here. Some authors indicated that the permeability coefficient of SRM decreases first and then increases with the increase of rock content; when the rock content was 40% (by volume or mass), the permeability coefficient achieved a significant minimum [8,10]. Zhou et al. [13] showed that the permeability coefficient of SRM increases with the increase in rock content.

Our test results are related to the permeability of rock and soil matrix. We assume that the rock is impermeable and that water flows through the pores between fine particles. When the rock content is 10%, the soil content is high, and the rocks are dispersed in the soil. Because the rock is impermeable, the increase in rock content is equivalent to reducing the cross-sectional area of the water flow, so when the rock content is 30%, the permeability coefficient decreases slightly. When the rock content is more than 30%, part of the argillaceous rocks are crushed during the compaction process, which reduces the pores formed between the rocks and keeps the porosity constant (see Table 1). However, the pores formed between the rocks in a local area will still exist, especially when the rock content is 70% and 90%, so the permeability coefficient increases significantly. At this time, the seepage flow in the embankment dam will increase, resulting in the increase in uplift pressure, and the weight of the dam is offset so as to reduce the anti-sliding force of the slope of the embankment dam and affect the stability of the dam.

We attribute these different results to differences in materials, compaction methods, and loading conditions. After compaction, confining pressure can further increase the compactness of soil and rock and reduce the porosity of the soil matrix [8]; The diameter of clay is smaller than that of silty clay, and continuously graded clay can better fill the pores between rocks [10]. These two conditions will lead to a significant decrease in the permeability coefficient. Zhou et al. [13] did not use the optimum water content in the compaction, which may lead to an increase in porosity with rock content at the same compaction, resulting in an increase in permeability coefficient with increasing rock content.

We have to point out that we do not obtain the permeability coefficient at 40% rock content (by mass), but according to the trend of the permeability coefficient we obtained, it is possible to reach the minimum value when the rock content is 40%. In addition, some authors define rock content by volume percentage, in which, when the permeability coefficient reaches the minimum value, the rock content (by volume) is 40%. If the volume percentage of 40% is converted to the mass percentage, in the following analysis, we know that the mass percentage is more than 40%. We assume that the volume of SRM after compaction is 1 (m^3^), the volume percentage *p_r_* = 40%, and the mass percentage *q_r_* can be expressed by

(10) $$p_{r}=\frac{V_{r}}{V}$$

(11) $$q_{r}=\frac{m_{r}}{m}$$

where *V_r_* (m^3^) is the volume of rock, *V* = 1 (m^3^) is the volume of SRM, *m_r_* (kg) is the mass of rock, and *m* (kg) is the mass of SRM. The ratio of mass percentage to volume percentage is

(12) $$\frac{q_{r}}{p_{r}}=\frac{m_{r}}{m}\times \frac{V}{V_{r}}=\frac{\rho_{r}}{\rho }$$

where *ρ* (kg m^−3^) is the density of SRM and *ρ_r_* (kg m^−3^) is the density of rock. The *ρ* can be calculated by:

(13) $$\rho =\frac{m}{V}=m_{r}+m_{s}=0.4\rho_{r}+0.6\rho_{s}$$

where *m_s_* (kg) is the mass of soil and *ρ_s_* (kg m^−3^) is the density of soil. Therefore, the ratio of mass percentage to volume percentage is:

(14) $$\frac{q_{r}}{p_{r}}=\frac{\rho_{r}}{0.4\rho_{r}+0.6\rho_{s}}=\frac{1}{0.4+0.6\frac{\rho_{s}}{\rho_{r}}}$$

Because the soil is the product of rock weathering, so the *ρ_s_* < *ρ_r_*, we get the *q_r_* > *p_r_* = 40%. A large number of research results show that the permeability coefficient increases when the rock content (by mass) exceeds 50% [10,46,47,48]. When the rock content (by volume) is lower than 25%, the rock floats in the soil, and the skeleton of rock has not been formed [13]. After the rock content is more than 25% (and is converted to a mass percentage greater than 25%), the soil fills the pores between the rocks, and the rock reduces the cross-sectional area of the water flow [13]. The permeability coefficient begins to decrease with the increase of rock content when rock content is more than 25%. Therefore, we infer that the minimum permeability coefficient of SRM is obtained between 30% and 50% of the rock content (by mass).

### 4.3. Influence of Rock Content on Streaming Potential Coupling Coefficient

The streaming potential coupling coefficient first increases and then decreases with the increase in rock content. This result is analyzed by the surface conductivity and flow state. Firstly, we calculate the surface conductivity, which is used to explain the relationship between structure and streaming potential coupling coefficient at low salinity [24,26]. Here, the surface conductivity is neglected because it is smaller than the solution conductivity (see Table 1).

**Figure 8:**
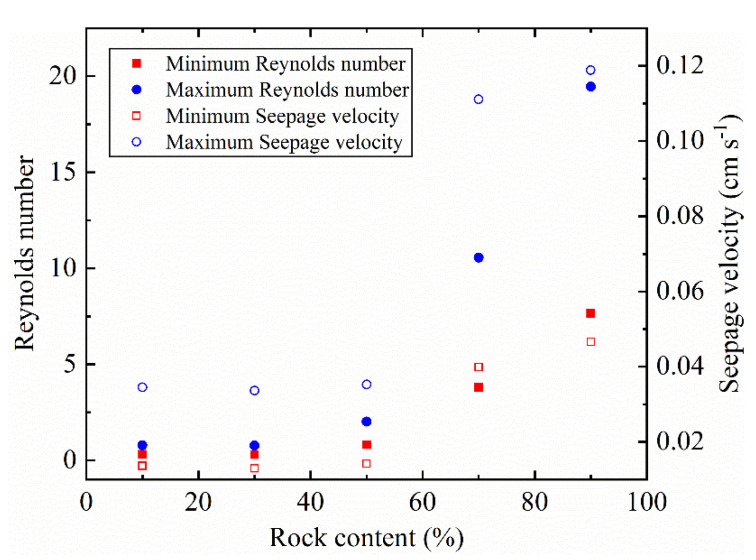
Reynold numbers and seepage velocity of SRM with different rock contents.

Secondly, the flow state of the SRM with different rock contents is analyzed. The zeta potential is independent of the structure, and the streaming potential coupling coefficient is controlled by the HS equation to maintain a constant in viscous laminar flow. However, the different rock content of the SRM under the same water flow conditions will lead to a different flow state. We calculate the maximum Reynolds number and the minimum Reynolds number to get the flow regime with different rock content at different water levels. The Reynolds number is obtained by [49]:

(15) Re=ρfUdμ

where *ρ_f_* (kg m^−3^) is the bulk density of the pore water, *U* (m s^−1^) is seepage velocity, and *d* (mm) is a certain length dimension of the porous medium. When the particle grading is well graded, we take *d* = *d*_50_; if the particle grading is poorly graded, we suggest *d* = *d*_70_; *d*_50_ and *d*_70_ is the particle sizes of 50% and 70% the total mass on the particle size distribution curve, respectively. When the rock content increases from 30% to 90%, the minimum and maximum Reynolds numbers gradually increase (see Figure 8). The minimum Reynolds numbers are more than 1 at rock contents of 70% and 90%. This means that the flow regime has changed [50]. When the flow state transitions from viscous laminar flow to inertial laminar flow, the streaming potential deviates from the HS equation [51]. The slope of the regression line between streaming potential and pressure difference decreases greatly when the fluid inertia (seepage velocity) is large. Therefore, when the rock content is increased from 30% to 90%, the streaming potential coupling coefficient decreases with the increase in rock content, while the streaming potential coupling coefficient shows an opposite trend when the rock content increases from 10% to 30%.

When the rock content exceeds 50%, the streaming potential decreases rapidly. This result can be explained by the reduction in the streaming potential coupling coefficient and the pressure difference according to Equation (1). As the main contribution of the self-potential signal, the streaming potential has an influence on the characteristics of the self-potential field distribution. Based on the relationship between streaming potential and rock content, we can use the self-potential method to reflect the change of rock content in SRM. This provides a new idea for us to understand the meso-structure of SRM. Further develop the array self-potential method [52] and combine it with a variety of geophysical exploration techniques [53,54,55], which would be helpful in order to achieve remote monitoring of structure of embankment dam.

## 5. Conclusions

In this paper, a self-designed apparatus is applied to investigate the streaming potential phenomenon of SRMs with different rock contents at 85% compactness and 0.01 mol L^−1^ salinity. The value of streaming potential and streaming potential coupling coefficient are recorded with increases in the rock content. The main conclusions are as follows:

(1) The permeability coefficient of SRM first decreases and then increases with the increase in rock content. The value of minimum permeability coefficient is determined by material, compaction method and loading condition. The reference range of rock content for obtaining the minimum permeability coefficient is 30 to 50%. Our results still need many experiments to verify. Considering more groupings within the range of 30% to 50% rock content will be an effective method to determine the minimum permeability coefficient.
(2) With the increase in rock content, the streaming potential coupling coefficient of the SRM increases until a larger value is reached at a rock content of 30%. As the rock content exceeds 30%, the streaming potential coupling coefficient decreases. The change in flow pattern leads to this result. The streaming potential phenomenon of other SRM materials with the change of rock content needs more experiments to confirm.
(3) The streaming potential decreases rapidly when the rock content exceeds 50%, which means that the self-potential signal will change significantly. By capturing this distribution feature of self-potential field, it shall be possible to build an early warning system for dam safety using the self-potential method.

## Figures and Tables

**Table 1:** Properties of SRM with different rock content.

Rock Content	10%	30%	50%	70%	90%
Porosity	0.32	0.30	0.30	0.31	0.30
Temperature (°C)	24	23	25	24	23
Electrolyte conductivity (S m^−1^)	0.089	0.088	0.087	0.091	0.089
Sample conductivity (S m^−1^)	1.35 × 10^−3^	1.33 × 10^−3^	1.36 × 10^−3^	1.45 × 10^−3^	1.48 × 10^−3^
Formation factor	66.86	67.02	65.16	63.54	61.32
Dynamic viscosity ^a^ (Pa s)	8.95 × 10^−4^	9.16 × 10^−4^	8.74 × 10^−4^	8.95 × 10^−4^	9.16 × 10^−4^
Relative permittivity ^b^	79	79	78	79	79
Zeta potential (mV)	−72.48	−74.92	−69.96	−48.34	−38.84
surface conductivity (S m^−1^)	1.24 × 10^−3^	1.25 × 10^−3^	1.59 × 10^−3^	1.41 × 10^−3^	1.69 × 10^−3^

^a,b^ Determined from Equations (13) and (12) in the reference [26].

## Data Availability

Not applicable.
